# Treat-to-Target in Systemic Lupus Erythematosus: Reality or Pipe Dream

**DOI:** 10.3390/jcm12093348

**Published:** 2023-05-08

**Authors:** Dina Zucchi, Chiara Cardelli, Elena Elefante, Chiara Tani, Marta Mosca

**Affiliations:** 1 Rheumatology Unit, Department of Clinical and Experimental Medicine, University of Pisa, 56126 Pisa, Italy; 2 Department of Medical Biotechnologies, University of Siena, 53100 Siena, Italy

**Keywords:** systemic lupus erythematosus, treat-to-target, remission, low disease activity, quality of life, treatment

## Abstract

Treat-to-target is a therapeutic approach based on adjustments to treatment at set intervals in order to achieve well-defined, clinically relevant targets. This approach has been successfully applied to many chronic conditions, and in rheumatology promising results have emerged for rheumatoid arthritis. For systemic lupus erythematosus (SLE), defining the most meaningful treatment targets has been challenging, due to disease complexity and heterogeneity. Control of disease activity, the reduction of damage accrual and the patient’s quality of life should be considered as the main targets in SLE, and several new drugs are emerging to achieve these targets. This review is focused on describing the target to achieve in SLE and the methods to do so, and it is also aimed at discussing if treat-to-target could be a promising approach also for this complex disease.

## 1. Introduction

Treat-to-target (T2T) is a therapeutic approach in which adjustments to treatment are made at set intervals in order to achieve a well-defined, clinically relevant target. T2T strategies include choosing a target and a method for measuring it, taking steps to achieve it, assessing the target at a pre-specified time point and changing the treatment if the target is not achieved [1]. The change in treatment does not necessarily have to be a switch of drugs but can be a change in lifestyle or an increase in the dose of previously introduced drugs.

The concept of T2T has been widely used in the treatment of chronic diseases such as diabetes, hypertension, hyperuricaemia and hyper-lipidaemia, using specific quantitative parameters as targets (glycated haemoglobin, blood pressure, uric acid and cholesterol levels, respectively), since in these patients the achievement of the targets can minimize organ damage and increase life expectancy [2].

The application of the T2T strategy to rheumatic diseases is more challenging, due to the complexity of the diseases and the absence of a specific or direct marker to assess disease activity. In clinical practice clinicians use composite scores that generally include not only biomarkers, but also physician’s assessed measures and patient-reported outcomes, and it is therefore difficult to identify a unique and ideal target. The T2T concept has been recently applied to rheumatoid arthritis (RA) with promising results; clinical remission, defined as the absence of signs and symptoms of significant inflammatory disease activity, was indicated by the European League Against Rheumatism (EULAR) recommendations as the primary target of the treatment strategy in RA, and low disease activity as an acceptable alternative therapeutic goal [3,4]. The efficacy of the T2T strategy in RA is supported by several clinical trials, including the FIN-RACo [5], the TICORA [6], the CAMERA [7] and the BeSt [8] study, confirming that this approach may improve the care of patients and provide useful guidance to healthcare professionals.

Systemic lupus erythematosus (SLE) is a more complex disease with respect to RA, due to the wide range of possible clinical manifestations, the relapsing–remitting course and the complexity of the composite scores used to assess disease activity.

In the T2T recommendations for SLE [9] different targets were identified, with particular emphasis on disease activity and damage prevention keeping the lowest glucocorticoid (GC) dosage and withdrawal if possible. In addition, the need to take into account the patient’s health-related quality of life (HRQOL) was underlined. The T2T recommendations in SLE are detailed in Table 1.

Although many targets have been identified in the T2T recommendations, at present the main available data on targets in SLE treatment concern remission, Lupus Low Disease Activity State (LLDAS) in non-renal and renal lupus and GC reduction.

The purpose of this review is to describe these targets to be achieved in SLE, the methods to do so and to discuss whether at present a T2T approach in SLE is possible.

## 2. Achievement of Remission and Low Disease Activity

Several definitions of remission have been developed, all of which include as main components the absence of clinical disease activity; treatment, particularly referring to GC doses; and, in some cases, serological activity [10,11,12,13,14]. Recently a large international task force (DORIS—Definition Of Remission In SLE) has developed a definition of remission in SLE [15,16] and the last updated criteria from this task force are reported in Table 2.

One of the most notable differences with respect to the previous criteria from the same Task Force [15] is that serological markers (anti-dsDNA, C3 and C4) were not included.

This is because, although some studies have shown that abnormalities or changes in serology predict flare or response to treatment, abnormal serology was not an independent predictor of damage, late morbidity or mortality in most of them [16].

Where remission cannot be reached, the lowest possible disease activity represents a target for disease activity control in SLE. Different definitions of low disease activity have been proposed [17]. Recently, the Asia–Pacific Lupus Collaboration group has developed and validated a definition of LLDAS [18], as detailed in Table 3, which has been largely applied in clinical practice as well as in randomized controlled trials (RCTs).

Remission and LLDAS prevalence varied among studies and cohorts. Remission is the ultimate goal in SLE, but it could be difficult to achieve and even more difficult is to maintain over time [19]. Remission has been reported in 2.5% [11] to 90.4% [20] of patients in the different cohorts with a notable increase over the years, despite in cases of more stringent definitions where remission was achieved by a lower percentage of patients [21]. Percentages of patients who achieved sustained remission (at least 5 years) are reported in Table 4.

LLDAS prevalence was generally higher than remission, being reached by more than 80% of patients in several cohorts [20,30]; in addition, LLDAS was maintained over time by 33.5% to 52.5% of patients (Table 5).

Reaching the targets of remission and LLDAS has proven to be linked to better outcomes in SLE, in terms of damage accrual, a reduction in the number of flares, GC withdrawal, better quality of life [34], reduced risk of cardiovascular disease [26], improved mortality [35] and also reduced direct healthcare costs [36].

One of the largest studies exploring the impact of achieving treatment targets on damage showed that reaching remission even as low as <25% at the time of follow-up and achieving LLDAS in 50% of follow-up visits led to a 50% reduction in damage accrual [37]. Several studies have also demonstrated that it is important to achieve remission as early as possible in the disease course (within one year from disease onset), to prevent early damage accrual and to prevent disease flares, to spare GC [38,39].

The attainment of treatment targets is not the only element to be considered. Growing evidence from the literature underlines that time spent in remission or LLDAS is a crucial point. So, it can be hypothesized that remission or LLDAS need to be a durable state to be considered a desirable treatment outcome [20]. In a cohort of Caucasian patients with SLE, two consecutive years appeared as the shortest duration of remission associated with a decrease in damage progression [22].

Data coming from different SLE cohorts confirmed that prolonged remission or LLDAS (defined as a 5-year consecutive period) are both associated with a lower risk of damage accrual, irrespective of other factors such as age, gender, racial group, serology or immunosuppressive treatment. In the LUMINA cohort, this protective effect was also shown on mortality, although statistical significance was not reached [14,24,40].

The definitions of both remission and LLDAS take into consideration ongoing GC treatment. Actually, GCs are responsible for much of the damage accrual, infections and premature mortality in SLE [41,42,43]. In this context, although reaching LLDAS is more frequent than remission, remission sounds intuitively preferable than LLDAS as it would probably lead to a lower GC burden over time.

The independent impact of different definitions of remission and LLDAS on damage accrual has been recently examined, for the first time, in a large multinational, multiethnic cohort (the Systemic Lupus International Collaborating Clinics (SLICC) inception cohort). Five mutually exclusive disease activity states were defined: remission off-treatment and on-treatment; low disease activity Toronto cohort (cSLEDAI-2K score of ≤ 2, without prednisone or immunosuppressants); and modified LLDAS (LLDAS definition without PGA) were compared to active disease. Achieving any of these possible targets was associated with a lower probability of damage accrual, even after adjusting for possible confounders and effect modifiers, highlighting the importance of treating-to-target in SLE. Moreover, in this cohort, a relatively high rate of remission was found, compared to LLDAS, thus encouraging the use of remission on- or off-treatment as the ideal target, with LLDAS being only an alternative target [44].

The association of remission and LLDAS and HRQOL is not unequivocal. Some studies have showed an association between remission or LLDAS achievement and better HRQOL in SLE patients, especially when a durable, stable remission is achieved [45,46]. Interestingly, major effects have been demonstrated on the physical component, whereas the mental component of quality of life seems to remain unchanged by remission [47]. However a recent study in a large Italian cohort has demonstrated that, although LLDAS is a satisfactory treatment target for the physician, it may not represent the ideal goal from the patient’s perspective, particularly when a low disease activity state “allows” the presence of ongoing arthritis and steroid therapy [48].

Therefore, it appears crucial to carry out a more comprehensive assessment of associated symptoms and conditions, such as fibromyalgia, mood disorders and fatigue [27,49]. In fact, the persistence of symptoms such as pain and fatigue, even when remission of SLE disease activity has been achieved, has largely emerged as an unmet need from patients’ perspectives [50,51].

## 3. Reaching Glucocorticoids Minimization and Withdrawal

It is well established that long-term GC use is associated in a dose-dependent manner with organ damage accrual including osteoporotic fractures, coronary artery disease, cataracts, avascular necrosis and stroke [52,53].

In a large SLE cohort it has been demonstrated that the current use of GCs at a dosage of 20 mg prednisone or more is associated with a five-fold increase in cardiovascular events [42]. Moreover, Ruiz-Irastorza et al. have demonstrated that with each increase of 10 mg per day of prednisone, there is an 11-fold increase in serious infections, in addition to an increased risk of avascular necrosis and the other numerous side-effects associated with exposure to supraphysiological doses of GCs [43].

Organ damage in SLE is associated with increased mortality [54]; therefore, according to the treat-to-target strategy and the EULAR recommendations, GC minimization and, when possible, complete GC withdrawal are considered important targets to be pursued [53,55]. However, GC tapering below 5 mg/day seems to be more difficult in older patients, in patients treated before 2000 and in cases of high disease activity and skin and musculoskeletal manifestations [56].The CORTICOLUP trial showed that the maintenance of long-term 5 mg prednisone prevents relapse [57]. In this RCT, patients in remission were randomized to GC withdrawal or maintenance; the proportion of patients experiencing a flare was significantly lower in the maintenance group as compared with the withdrawal group (4 patients vs. 17, *p* = 0.003). However, the majority of flares were mild–moderate. However, several real-life data suggest that GC discontinuation could be safe [58,59,60,61] in patients with long-term quiescent disease, and disease flares were not common in this subset of patients [58,59,60].

Although, with some caveats, therefore, the literature shows that GC withdrawal is feasible, particularly in patients with long-term remission or LLDAS.

## 4. Control of Lupus Nephritis

In the context of lupus nephritis (LN), we have more data available on the targets to be achieved and the timelines for achieving them. Recently, the EULAR recommendations for the management of LN have clearly defined specific goals of therapy [62].

In LN, the prediction of the long-term renal outcome at the early stages of the disease is of vital importance [63]. With this premise, adhering to a T2T strategy in clinical practice may facilitate the management and follow-up of LN patients, particularly when a clear target to be pursued is identified. Recently, the analyses of two important lupus trials, the MAINTAIN Nephritis trial [64] and the EuroLupus Nephritis Trial [65], have reported that proteinuria is the single best predictor of long-term (7 years) renal outcome in lupus patients, suggesting a possible use of proteinuria as a target to prevent renal damage in a T2T approach. Similarly, in a real-life situation, proteinuria at 12 months of follow-up was found to be the single best predictor of renal outcome at 7 years for an ethnically diverse group of patients with severe nephritis and a valid parameter for distinct histological classes, races, genders and anti-dsDNA [66].

A renal complete response, defined as proteinuria < 0.5–0.7 g/24 h with a glomerular filtration rate (GFR) normalization/stabilisation in 12 months from LN onset, is considered a clinically meaningful target to be achieved as it is associated with a good long-term renal prognosis.

However, in the meantime, at least an improvement in proteinuria should be obtained within 3 months in combination with a normalization/stabilization of GFR and a partial clinical response, defined as a reduction in proteinuria of at least 50%, should be achieved by 6 months. The time to reach the target could be extended for 6–12 months in patients with nephrotic-range proteinuria, to avoid premature treatment changes.

## 5. Do Available Therapies Help in Achieving Targets?

Until a few years ago, the therapeutic armamentarium for SLE included GCs, antimalarials, traditional immunosuppressive drugs and few biological drugs. These drugs are valuable aids in achieving targets; of note is how the response to placebo (plus standard of care) is above 36% for all primary endpoints in non-renal, non-neuropsychiatric SLE RCTs [67]. However, reaching therapeutic targets remains an unmet need in a considerable proportion of patients, highlighting the importance of developing new drugs that improve the disease outcomes.

In recent years, scientific advances have led to the development of new pharmacological agents and several trials have been conducted or are currently underway to evaluate their safety and efficacy in clinical practice [68].

According to the 2019 EULAR updated recommendations for the management of SLE, while GCs and hydroxychloroquine remain the milestones for the early treatment of non-renal SLE, the prompt initiation of immunosuppressive therapy in moderate-to-severe or refractory mild SLE should allow easier achievement of therapeutic goals [55]. It should be noted, however, that traditional synthetic disease modifying antirheumatic drugs (DMARDs) are often burdened with side-effects and their use, in the long-term, may contribute to damage accrual. Therefore, in order to minimize drug-related toxicity, a better control of disease activity, achieved in less time, allows a greater sparing of daily and subsequently cumulative GC dosing and an optimization of DMARD use.

### 5.1. Belimumab

Recent data have shown that belimumab, an anti-B lymphocyte stimulator (BlyS, also known as BAFF) monoclonal antibody, is useful in helping to achieve remission or LLDAS targets. Indeed, attainment of LLDAS at week 52 was significantly more frequent in patients on belimumab compared to placebo (12.5% vs. 5.8%, OR 2.32, *p* = 0.02 for BLISS-52; 14.4% vs. 7.8%, OR 1.98, *p* = 0.04 for BLISS-76) [69]. In particular, belimumab seemed to be more efficacious in reaching LLDAS and clinical remission in SLE patients without organ damage prior to starting treatment [70]. Although not always reaching statistical significance, RCTs on belimumab have also shown that this drug may help in reducing severe flares evaluated by the modified SLE Flare Index and has a steroid-sparing effect, being able to reduce the long-term damage accrual [71,72]. Post-hoc analyses of the BLISS-52 and BLISS-76 trials showed a greater response to belimumab in patients with elevated BlyS mRNA and/or protein expression at baseline [73], suggesting a potential role for biological biomarkers in patient stratification that may predict a better response to treatment and an easier achievement of targets.

### 5.2. Anifrolumab

Anifrolumab, a fully human IgG1κ monoclonal antibody targeting the subunit 1 of the type I interferon receptor (IFNAR1), proved to be a promising drug to reach the therapeutic goals. Post-hoc analysis of TULIP-1 and TULIP-2 phase III RCTs demonstrated that anifrolumab treatment was associated with earlier, more frequent (30.0% vs. 19.6% at week 52; OR 1.8, 95% CI 1.3 to 2.5, *p* = 0.0011) and more prolonged and sustained LLDAS attainment compared to placebo [74]. Moreover, anifrolumab was also associated with higher rates of DORIS remission (15.3% vs. 7.6% at week 52; OR 2.2, 95% CI 1.4 to 3.6, *p* = 0.0013) and with earlier and more sustained achievement of remission compared to placebo [74,75]. With regard to steroid therapy, this new biotechnological drug demonstrated the possibility of achieving a sustained reduction in the daily dosage of GCs, thus potentially reducing the overall damage accrual [76]. Subgroup analysis of pooled TULIP data proved the greatest difference from placebo in SLE patients with a high IFN gene signature [77].

### 5.3. New Drugs for Lupus Nephritis

Recently, the approval of two new treatments for LN, voclosporin and belimumab, opened up new possibilities in the development of a T2T strategy [78]. Voclosporin, a novel calcineurin inhibitor, is the first oral therapy approved for the treatment of active LN (in combination with standard of care), based on the AURA-LV and AURORA-1 trials, in which the proportion of patients achieving complete renal response was significantly higher with add-on voclosporin compared to placebo (40.8% vs. 22.5%; OR 2.65, 95% CI 1.64–4.27, *p*< 0.0001) [79,80].

Overall, the new drugs available represent additional options to the standard of care to allow a T2T strategy for SLE patients in view of their efficacy in controlling disease activity and their steroid-sparing effect.

## 6. Discussion: From Disease Targets to “Treat-to-Target”

The concept of T2T seems to be less applicable in SLE than in other chronic non-rheumatological and rheumatological conditions due to the heterogeneity and complexity of the disease, and the absence of a unique marker of disease activity makes it challenging; consequently, it appears unrealistic to identify a single and effective treatment target for SLE patients. However, the implementation of T2T remains a major goal in SLE and aims not only to achieve the best possible control of disease activity, but also to prevent damage accrual and improve patients’ quality of life.

The identification of different molecular signatures in the pathophysiology of such a complex disease [81] arouses interest in precision medicine and suggests that the stratification of patients according to certain markers of disease activity and flare predictors (e.g., BlyS levels, IFN signature) might provide advantages in achieving T2T, although to date it is still not widely practicable in routine patient care.

To make T2T possible in clinical practice, at least two objectives must be achieved: establish practical and achievable targets and develop therapeutic options that can realistically allow these outcomes to be achieved at established time points.

Overall, there is a general agreement on the fact that the control of disease activity with the achievement of remission or at least LLDAS should be considered as the main targets to be reached with treatment in SLE [55]. Real-life data showed that remission is an achievable target in many SLE patients [14,58,59,60,61]. However, prolonged remission is less frequent, due to recurrent flares, persistent disease activity or the inability to taper GCs.

Another challenge is when targets have components that are in conflict, and discrepancy between cSLEDAI and PGA was previously reported by Saccon F et al. after testing different definitions of remission in a large multicentre cohort [25]. Adding PGA < 0.5 to cSLEDAI appears to have led to loss of remission in a relevant proportion of patients, without significant improvement in its predictive value against damage. The authors pointed out that additional treatment is not always necessary in patients with cSLEDAI = 0 despite PGA ≥ 0.5, and therefore adding PGA < 0.5to cSLEDAI = 0 may lead to overtreatment when a T2T approach is adopted.

Despite the targets seeming clear, we are currently far from an application of the T2T approach in clinical practice.

Indeed, some barriers could be envisaged in the implementation of T2T SLE; the first problem relies on the fact that there is a lack of knowledge on how disease targets should be achieved.

For instance, a 6-monthly interval has been proposed for monitoring disease activity in the overall T2T strategy for SLE [82], but it is not clear if it is the best timing.

Thus, to apply T2T in SLE with remission or at least LLDAS as principal targets, some questions need to be answered such as: Which is the time necessary to achieve the target? Is it different with different drugs or disease manifestations? How frequently should we assess our patient to establish if the target is achieved or if we should change the treatment? What should be our boundary point between the need to control the disease and avoid drug toxicity?

Given the evidence of the benefits of optimising the management of GC therapy and the need to minimize the dose, further studies comparing LLDAS and remission would be necessary, considering the higher GC dose allowed in the LLDAS definition. Thus, setting the ambitious goal of aiming for remission rather than LLDAS could help to minimize the drug-related toxicity. However, certain comorbidities and the lack of a “perfect treatment” continue to make remission an unrealistic target for several patients.

A strategy to be used to facilitate the implementation of T2T should include the involvement of the patient in decision making, also with a view to improving adherence to therapy, which is another possible concomitant cause of treatment failure. The percentage of non-adherent patients is described to be up to 75%, while up to 33% of patients discontinue therapy after 5 years [83], underlining the importance of the implementation of communication to increase patient knowledge about the disease and the benefits of prescribed therapies.

Lastly, it has yet to be demonstrated that a T2T strategy is beneficial in SLE, in terms of improving clinically relevant outcomes, improving quality of life and saving health care resources.

A protocol for the first trial aimed at investigating if the T2T strategy in SLE minimises damage accrual and improving quality of life was recently published [84]. This study will perform a comparison of remission and LLDAS in order to assess the benefit/risk ratio and avoid unrealistic targets or timeframes for achieving this.

To give further detail, patients will be equally randomized in three arms: two intervention arms (LLDAS and remission), in which patients will be treated to target, and a control group (standard of care). In the intervention arms, in cases of patients not on target, visits will be performed every six weeks with treatment adjustment until the target is reached and maintained, while patients on target will be evaluated every 12 weeks. In the standard of care arm, patients will be assessed every three or six months, based on the physician’s judgement. At the end of the study (120 weeks), change in damage accrual and quality of life will be the major outcomes.

## 7. Conclusions

Based on the available data, the application of T2T in SLE seems possible. In many referral centres this strategy is probably already applied in clinical practice, but there are no standardized protocols. As more and more patients are achieving a better disease control in recent decades, the implementation and application of T2T could further improve SLE patient management.

## 8. Future Directions

The efficacy of the T2T approach in SLE needs to be demonstrated with validated studies, and the above-mentioned trial proposed by Mucke J et al. [84], in association with real-life data, will help to allow the application of the T2T concept, with the aim of providing significant benefits to SLE patients.

## Figures and Tables

**Table 1:** T2T recommendations [9].

1.	The treatment target of SLE should be remission of systemic symptoms and organ manifestations or, where remission cannot be reached, the lowest possible disease activity, measured by a validated lupus activity index and/or by organ-specific markers.
2.	Prevention of flares (especially severe flares) is a realistic target in SLE and should be a therapeutic goal.
3.	It is not recommended that the treatment in clinically asymptomatic patients be escalated based solely on stable or persistent serological activity.
4.	Since damage predicts subsequent damage and death, prevention of damage accrual should be a major therapeutic goal in SLE.
5.	Factors negatively influencing health-related quality of life, such as fatigue, pain and depression should be addressed, in addition to control of disease activity and prevention of damage.
6.	Early recognition and treatment of renal involvement in lupus patients is strongly recommended.
7.	For lupus nephritis, following induction therapy, at least 3 years of immunosuppressive maintenance treatment is recommended to optimize outcomes.
8.	Lupus maintenance treatment should aim for the lowest glucocorticoid dosage needed to control disease, and if possible, glucocorticoids should be withdrawn completely.
9.	Prevention and treatment of antiphospholipid syndrome (APS)-related morbidity should be a therapeutic goal in SLE; therapeutic recommendations do not differ from those in primary APS.
10.	Irrespective of the use of other treatments, serious consideration should be given to the use of antimalarials.
11.	Relevant therapies adjunctive to any immunomodulation should be considered to control comorbidity in SLE patients.

**Table 2:** DORIS definition of remission.

DORIS Definition of Remission
Clinical SLE Disease Activity Index (cSLEDAI) = 0
Physician’s global activity (PGA) (scale 0–3) score < 0.5
Irrespective of serology
The patient may be on antimalarial, low-dose glucocorticoids (prednisolone < 5 mg daily) and/or stable immunosuppressive drugs including biologics

**Table 3:** LLDAS definition.

LLDAS Definition
SLEDAI 2000 (SLEDAI-2K) score ≤ 4, with no activity in major organ system (including renal, central nervous system, cardiopulmonary, vasculitis and fever) an no haemolytic anemia or gastrointestinal activity
No new features of lupus disease activity (according to SLEDAI-2K) compared with the previous assessment
SELENA SLEDAI-PGA (scale 0–3) score ≤ 1
Current prednisolone (or equivalent) dose ≤ 7.5 mg daily
Well-tolerated standard maintenance doses of immunosuppressive drugs and approved biological agents

**Table 4:** Sustained remission.

Reference	Definition of Remission	Duration of Remission	Number of Patients	Percentage of Remitted Patients
Zen et al. [14]	Zen et al., 2015	≥5 years	224	37.4%
Zen et al. [22]	Zen et al., 2015	≥5 years	293	38.6%
Mok et al. [23]	van Vollenhoven et al., 2017	≥5 years	769	8.3%
Tsang et al. [24]	Zen et al., 2015	≥5 years	117	32.5%
Saccon et al. [25]	Saccon et al., 2020	≥5 years	646	16.6%, 12.4%
Fasano et al. [26]	Zen et al., 2015	≥5 years	294	44.5%
Tani et al. [20]	van Vollenhoven et al., 2017	≥5 years	115	21.7%
Margiotta et al. [27]	Zen et al., 2015	≥5 years	136	39%
Ruiz-Irastorza et al. [28]	van Vollenhoven et al., 2017	≥5 years	173	50%
Nikfar et al. [29]	van Vollenhoven et al., 2017	≥5 years	193	59.6%

Definition of remission: Zen et al., 2015 [14]: cSLEDAI = 0, treatments allowed: antimalarials, stable immunosuppressant therapy, 1–5 mg prednisone (PDN) daily; van Vollenhoven et al., 2017 [15]: DORIS definition; Saccon et al., 2020 [25]: cSLEDAI = 0 + PDN ≤ 5 mg/day, cSLEDAI = 0 + PDN ≤ 5 mg/day + PGA < 0.5.

**Table 5:** Sustained LLDAS.

Reference	Definition of LLDAS	Duration of LLDAS	Number of Patients	Percentage of LLDAS Patients
Zen et al. [31]	Franklyn et al., 2016	≥5 years	293	37.2%
Tani et al. [20]	Franklyn et al., 2016	≥5 years	115	36.5%
Babaoglu et al. [32]	Franklyn et al., 2016	≥50% of the observation time	2228	52.5%
Sharma et al. [33]	Franklyn et al., 2016 (but excluding PGA value, not available for the cohort)	at least half of the follow up time (median duration 125 months)	206	33.5%

Definition of LLDAS: Franklyn et al. [18].

## Data Availability

Not applicable.
